# Raman Spectroscopy of Conical Intersections Using Entangled Photons

**DOI:** 10.1021/acs.jpclett.3c02852

**Published:** 2024-02-13

**Authors:** Deependra Jadoun, Zhedong Zhang, Markus Kowalewski

**Affiliations:** † Department of Physics, Stockholm University, AlbaNova University Center, SE-106 91 Stockholm, Sweden; ‡ Department of Physics, City University of Hong Kong, Kowloon, Hong Kong SAR; § Shenzhen Research Institute, City University of Hong Kong, Shenzhen, Guangdong 518057, China

## Abstract

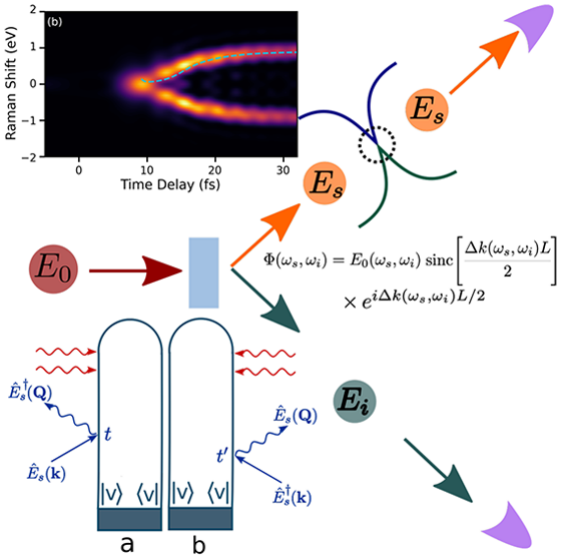

Ultrafast Raman spectroscopy with attosecond pulses in the extreme ultraviolet and X-ray regime has been proposed theoretically for tracking the non-adiabatic dynamics of molecules in great detail. The large bandwidth of these pulses, which span several electronvolts within a couple of femtoseconds, provides a unique tool for tracking non-adiabatic phenomena. However, spectroscopy with classical light is limited by the time–bandwidth product of the probe laser pulse. In this work, we theoretically investigate an ultrafast Raman spectroscopy scheme that utilizes pairs of entangled photons. Our model simulations demonstrate that the dynamics in the vicinity of a conical intersection can be resolved with unprecedented resolution in the time and frequency domain.

Photochemical processes that exhibit non-adiabatic dynamics originating from conical intersections (CIs)^1,2^ are ubiquitous in nature. CIs are believed to play an important role in processes such as vitamin D generation,^3,4^ damage to human DNA due to ultraviolet (UV) radiation and its repair,^5^ and photoinduced isomerization.^6,7^ In the vicinity of a CI, the Born–Oppenheimer approximation (BOA) breaks down and the electronic and nuclear degrees of freedom become strongly coupled.^8−10^ This process is commonly observed as an ultrafast nonradiative decay of excited electronic states. A detailed understanding of such ultrafast decay channels is essential for improving our understanding of photochemical processes in nature.

The primary obstacle in probing a CI by means of time-resolved spectroscopy is the requirement of adequate temporal and spectral resolutions of probe pulses. The population transfer near a CI can take place within a few femtoseconds. The associated energy gap between electronic states may cover several electron volts within the same time frame. Spectroscopic techniques that use optical pulses to probe these processes are thus limited to indirect measurements of a CI.^11−13^ Experimental^14−19^ and theoretical^20−26^ efforts have been dedicated to detecting the presence of CIs in molecules using ultrashort laser pulses. However, to directly detect a CI, both temporal and spectral resolutions are required. For detection processes that are linear in the intensity of the probe pulse, time and frequency are Fourier-limited. This can be partially overcome by using multidimensional probe schemes, which are based on two independent time variables.^22,27^

Quantum states of light provide a different avenue for increasing the time–bandwidth limit. Entanglement and nonclassical statistics of photons create a new parameter space and provide new control knobs. Probe schemes based on entangled photons have been shown to be promising for the study of ultrafast electron dynamics and CIs. Entangled photons allow for simultaneous resolution in the time and frequency domains, where classical light is bound to the Fourier limit. Such a quantum advantage has been explored in recent theoretical studies of photochemical reactions of molecules, which included the non-adiabatic dynamics monitored from two-photon absorption.^29^ Femtosecond coherent Raman spectra using entangled photons were reported recently as a proof-of-principle demonstration of enhanced resolutions for monitoring fast molecular dynamics.^30,41^ However, for molecular systems with a CI, there has not yet been a conclusive study, and it is still an open issue.

In this paper, we show quantum femtosecond Raman spectra for the detection of electronic coherences as they are generated in the vicinity of CIs. Entangled photons are used to scatter molecular excited states, which imprint the electronic coherence created at the CIs onto the signal. We demonstrate that the entangled photons allow for a greater time–frequency resolution than can be achieved with classical laser pulses. Our work provides a new scheme of optical techniques as versatile tools for exploring photochemical processes and reactivity.

We use entangled photons that are created by using a spontaneous parametric down-conversion (SPDC) process, in which a light pulse passes through a birefringent crystal and breaks into two entangled photons. The photons are entangled in time and frequency due to the law of conservation of energy, such that the sum of the frequency of the entangled photons is equal to the center frequency of the SPDC pump (ω_0_). The two entangled photons are directed into the s and i arms of the setup, as shown in panels a and b of Figure 1. The photon in the s arm with a frequency of ω_s_ interacts with the molecules, while the photon in the i arm with a frequency of ω_i_ propagates freely as a reference to the s arm photon. The entangled photon pair has the wave function^30^
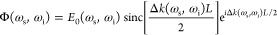
1where the *E*_0_ field is given by the SPDC pump with central frequency ω_0_.  provides the phase matching, where *Δk* represents the wave vector mismatch and *L* is the length of the SPDC crystal. *T*_s_ (*T*_i_) represents the delay between the SPDC pump and the photon in the s (i) arm of the setup. We consider, hereafter, the optimal entanglement between the two photons by setting *T*_s_ equal to *T*_i_.

**Figure 1:**
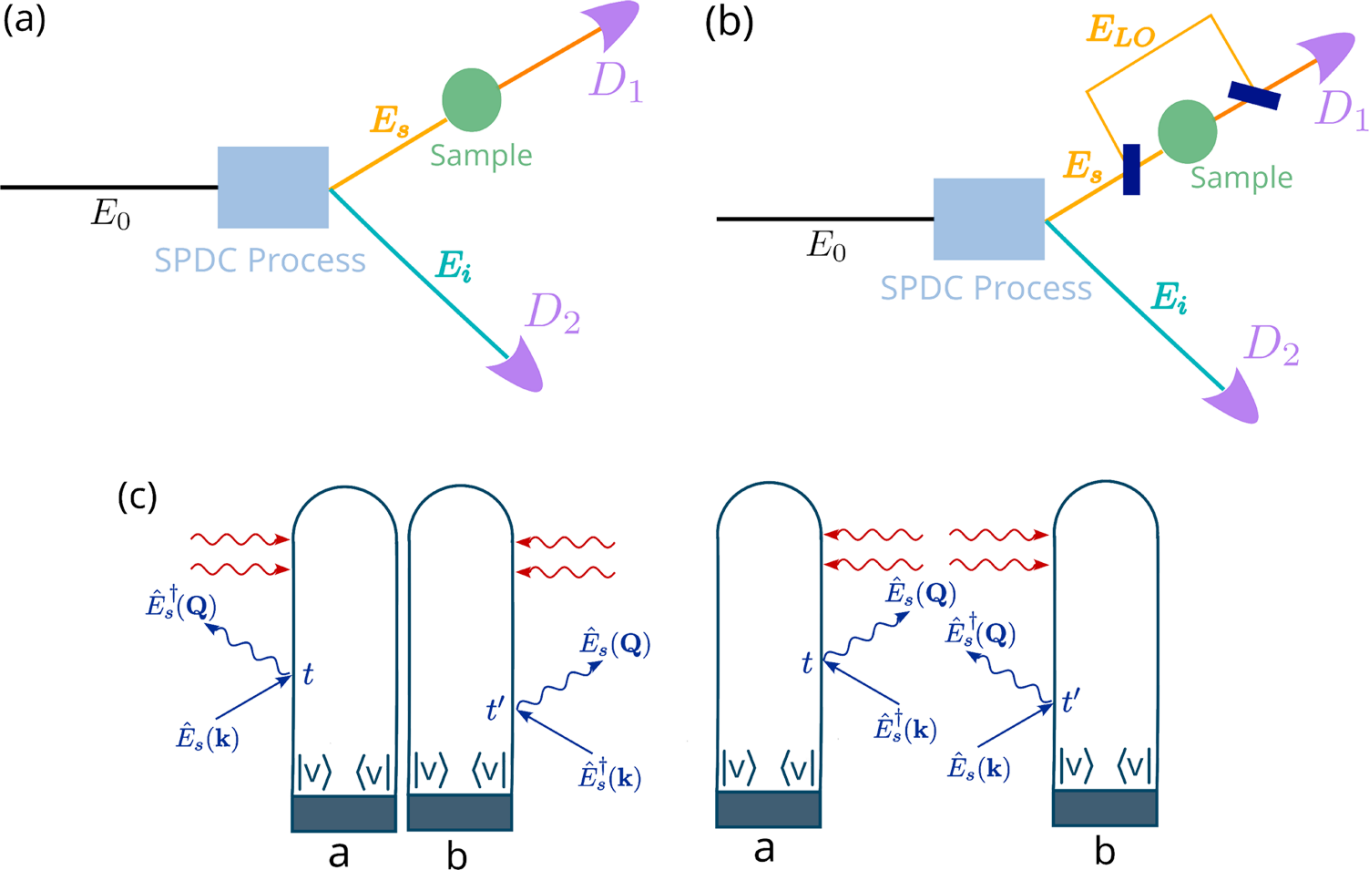
Schematic for coincidence detection setups for Raman signals using entangled photon pairs. (a) Homodyne detection setup. (b) Heterodyne detection setup. In both setups, photons are detected in coincidence at the end. In the heterodyne detection setup, the photon in the s arm is combined with a LO photon using interference after interacting with the sample. (c) Loop diagrams corresponding to the Raman process. The red arrows at the top of the loop diagrams represent the detection of the entangled photons in the s and i arms of the setup.

The coincidence-counting detection of the entangled photons in the s and i arms of the setup is considered in our study. Coincidence detection yields a high signal-to-noise ratio^31^ and allows for the utilization of the frequency entanglement between the photons. A frequency gate in the detector in the i arm of the setup is used to observe photons of a specific frequency, which fixes the frequency of the photon in the s arm as well due to frequency entanglement such that ω_s_ = ω_0_ – ω_i_. A spectrometer is used in the s arm detector to record the Raman shift in the photon due to the interaction with molecules. The temporal resolution is governed by the width of the SPDC pump pulse, while the spectral resolution is controlled by the propagating time of photons inside the nonlinear crystals that are subject to the photon dispersion and crystal geometry.

Two detection schemes for the Raman signal, i.e., homodyne and heterodyne as depicted in panels a and b, respectively, of Figure 1, interest us. The heterodyne-detected Raman signal is essential for revealing the phase of the electron dynamics. First, we consider the homodyne-detected Raman signal that derives from intensity-correlated measurement of photons and the Raman interaction (*N* molecules). It reads
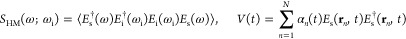
2where *E*_s_ is the electric field of the s arm photons with frequency ω_s_ and *E*_i_ is the electric field for the reference photon in the i arm with frequency ω_i_. α_*n*_(*t*) represents the polarizability tensor responsible for Raman transitions. The expected value in eq 2 is over the full wave function of molecules and fields, which naturally accounts for the molecule–light interaction. Using eq 1 and time-dependent perturbation theory with respect to the interaction *V*(*t*) in eq 2, one can calculate the homodyne-detected Raman signal with entangled photons^30^
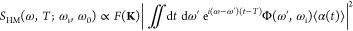
3for *N* identical molecules [α_*n*_(*t*) ≡ α(*t*)], where *T* is the pump–probe delay. *F*(**K**) is a form factor that may reveal the molecular crystalline structure. For identical molecules, it yields
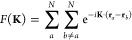
4where **r**_*a*_ and **r**_*b*_ represent the position vectors of identical molecules *a* and *b*, respectively. Here **K** is the wave vector of emitted photons. The form factor requires that the probe pulse is in the soft X-ray regime such that |**K**|*l* ≈ 2π, where *l* is the sample length. For pulses with wavelengths much larger than those of X-rays, the form factor takes a constant value because |**K**|*l* ≪ 2π, and the crystalline structure of the sample cannot be included in the signal. Additionally, the form factor in eq 4 depends on the relative positions of identical molecules and their orientation with respect to the probe field. The form factor takes a value of *N*(*N* – 1) for a sample that exhibits long-range order and perfect phase matching. Molecular samples in the condensed phase allow for such an observation. For molecules in the liquid phase, the form factor needs to be computed on the basis of the range of the order of molecules in the sample using polarization directions and relative positions.^32^ The ro-vibration relaxation in such samples comes into effect at a time scale of ∼10 ps, i.e., much longer than the time scale of interest (∼100 fs). Note that for the molecules in the gas phase the signal will disappear due to the random orientation of molecules, which results in the lack of an order of molecules in the sample.

The heterodyne-detected signal is obtained from the interference between the transmitted photons of the sample and the local oscillator (LO) field that carries an additional phase ϕ controlled by the optical path (see Figure 1b). This can be used to extract the electronic phase information, which is lost in the homodyne scheme. With *E*_lo_ being the local oscillator field with a phase difference of ϕ to the s arm photons, the calculations proceed as usual for the heterodyne-detected signal , and we find the heterodyne-detected Raman signal with entangled photon pairs
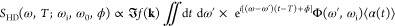
5where *f*(**K**) = ∑_*a*_^*N*^ exp(*i***K**·**r**_*a*_). Note that the heterodyne signal scales linearly with the number of molecules, *N*, because of the interference of the pathways.^30^

The non-adiabatic nuclear dynamics in a molecular model system with a CI is studied using the presented Raman technique. The nuclear dynamics can be initiated by using a pump pulse that excites the system from the ground state to the excited state. The molecular Hamiltonian in the presence of a pump pulse can be written as
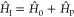
6where *Ĥ*_0_ represents the bare molecule Hamiltonian and *Ĥ*_P_ represents the interaction Hamiltonian of the molecule with the pump pulse. The bare molecule Hamiltonian that governs the nuclear wave function dynamics and coupling of electronic states can be expressed as
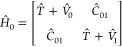
7where *T̂* = −(ℏ^2^/2*m*_r_)∇^2^ represents the kinetic energy operator, where ∇ is the gradient operator for the reaction coordinates and *m*_r_ = 30 000 au represents the reduced mass of the system. The potential energy surfaces (PESs) for the two electronic states are represented by *V̂*_0_ and *V̂*_1_, and *Ĉ*_01_ is the diabatic coupling between the electronic states. The pump pulse interaction Hamiltonian can be written as
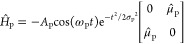
8where *A*_P_, ω_P_, and σ_P_ represent the amplitude, frequency, and pulse width of the pump pulse electric field, respectively. The transition dipole operator between the electronic states is given by μ̂_P_.

Figure 2a shows the one-dimensional diabatic energy curves of electronic states *V*_0_ and *V*_1_ that exhibit a CI. The two electronic states are coupled via diabatic couplings in the region of the intersection. The CI between the two states can be visualized by transforming the electronic states from the diabatic basis to the adiabatic basis, as shown in Figure 2b. The presence of a CI requires at least two degrees of freedom in a molecule, and hence, the electronic states in the used model system depend on two reaction coordinates. A molecule can have multiple degrees of freedom that operate on a time scale similar to that of the CI, and thus, all of the relevant reaction coordinates must be incorporated into the quantum dynamics simulations. The analytical expressions used to construct the electronic states and the diabatic couplings can be found in the Supporting Information.

**Figure 2:**
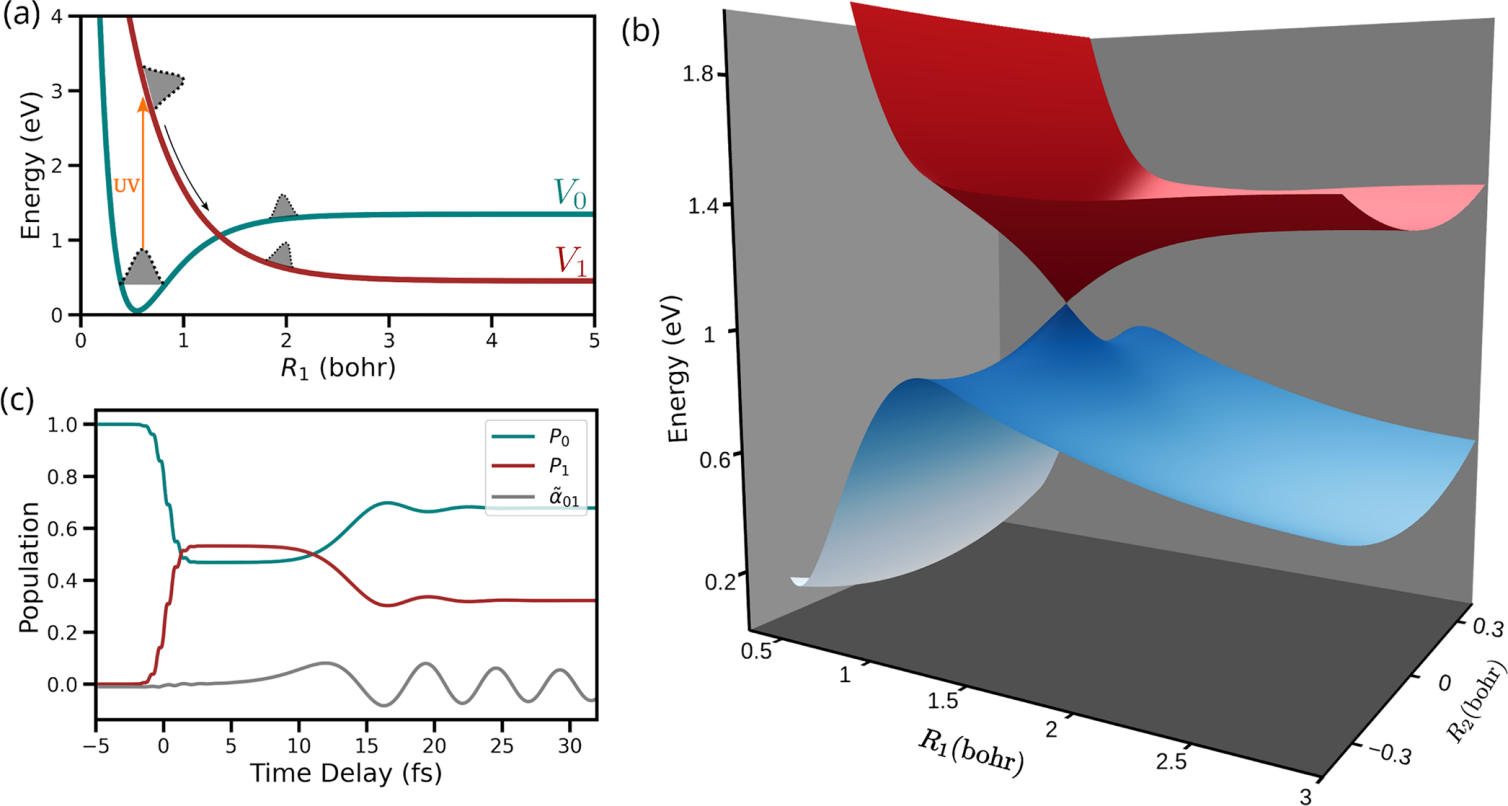
Overview of the model system and the CI. (a) One-dimensional diabatic potential energy curves for the *V*_0_ and *V*_1_ electronic states that exhibit a CI. (b) Adiabatic PESs showing the CI between two electronic states. (c) Population dynamics of the *V*_0_ state (*P*_0_) and the *V*_1_ state (*P*_1_), along with the Raman electronic transition, α̃_01_.

An ultraviolet (UV) pulse excites the system to the *V*_1_ state starting from the *V*_0_ state, which results in a nonstationary nuclear wave packet that passes through the CI. The population dynamics in the two electronic states following the UV pulse excitation are shown in Figure 2c. The dynamic nuclear wave packet in the *V*_1_ state reaches the CI by ≈10 fs, and population transfer takes place. Approximately 30% of the total population is transferred from the *V*_1_ state to the *V*_0_ state via the CI.

The Raman signal depends on the molecular polarizability matrix. For a specific direction, the polarizability operator takes the following form
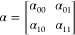
9where the diagonal elements (α_00_ and α_11_) give rise to the vibrational coherence features and the off-diagonal elements (α_01_ and α_10_) give rise to the electronic coherence features in a Raman signal. The summation of all of the polarizability elements gives a vibronic coherence contribution to a Raman signal. In general, elements in the polarizability matrix are not constant with respect to the reaction coordinates and depend on the energies of the core-hole state along with the transition dipole moment between the valence and core-hole states. See ref (33) for information about calculating polarizability from the PESs and transition dipole moments obtained using electronic structure calculations. For the sake of simplicity, we use constant polarizability operators that correspond to the vibrational coherence and antisymmetric step function as the polarizability operator for the electronic contribution. Consequently, the total Raman electronic transition, which shows features similar to those of the electronic coherence, is given by the following expression for α_10_ = α_01_
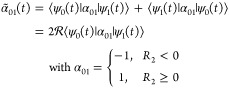
10where ψ_0_ and ψ_1_ represent ground and excited state nuclear wave functions that depend on nuclear coordinates *R*_1_ and *R*_2_, respectively. We assume an antisymmetric function with respect to *R*_2_ to define the Raman electronic transition via transition polarizability α_01_. Combined with the *R*_2_ antisymmetric diabatic couplings, this will result in a time-dependent Raman electronic transition, α̃_01_(*t*), which corresponds to the electronic coherence generated at the CI.^34^ Note that due to the required time and bandwidth requirements, the photon energies are in the X-ray regime. Entangled photon generation in the X-ray regime has been demonstrated experimentally.^35−39^

The time-dependent Raman electronic transition, α̃_01_(*t*), calculated using eq 10 is an oscillatory function, as shown by the gray curve in Figure 2c. The oscillation frequency is dependent on the difference in energy between the states that form the superposition. The result is a slow oscillation in α̃_01_ when the molecule reaches the CI, which accelerates as the difference in energy between the two electronic states increases over time. A classical broadband probe can be used to time the emergence of increasing α̃_01_(*t*) and can be seen as a direct signature of the CI. Figure 3a shows the homodyne-detected Raman signal constructed using eq 3 for a classical broadband probe, with a spectral width σ_f_ of 1.5 eV and a temporal width σ_t_ of 0.44 fs, for the electronic contribution to the Raman signal. Note that using a *T*_s_ of 0 fs in eq 3 eliminates the entanglement between the photons and yields a signal for a classical laser pulse. An oscillatory signal, proportional to α̃_01_(*t*), appears around 10 fs, which is caused by passage through the CI and the generated electronic coherence. The oscillations in the signal follow the magnitude of α̃_01_(*t*) shown in Figure 2c by the gray curve. Note that the time-dependent difference in energy between the involved states is not visible in the classical Raman signal due to the poor spectral resolution provided by the classical broadband probe pulse (Figure 3a). However, the dynamic energy separation between the electronic states can be extracted from the oscillating signal via a Wigner spectrum analysis. A classical narrow-band probe pulse can be used to follow the evolving electronic state separation in the Raman shift. However, this results in a poor temporal resolution, thus restricting the precise timing of the CI. Entangled photons can be used rather than a classical pulse to achieve a simultaneously high temporal and spectral resolution. The temporal resolution depends on SPDC pump field *E*_0_, and a femtosecond or subfemtosecond pulse should be used to study the nuclear dynamics near a CI. On the contrary, the spectral resolution is controlled by the delay between SPDC pump and entangled photons such that *T*_s_ = *L*/*v*_s_, where *L* is the crystal length and *v*_s_ is the group velocity of the photon in the s arm. For the used model system, the optimal delay between the SPDC pump and entangled photons (*T*_s_) was found to be 10 fs, which is comparable to the duration of the passage through the CI. The Raman signal for the entangled photon pair, for which σ_f_ = 1.5 eV, *T*_s_ = 10 fs, and ω_0_ – ω_i_ = 7.77 eV, is shown in Figure 3b. Here, a much higher spectral and temporal resolution is achieved when compared to the Raman signals generated with classical light. The passage of the CI becomes evident by the signal generated at ∼10 fs with zero Raman shift and subsequent Raman shifts to higher frequencies with an increase in the delay. The Raman signal shows a trend following the blue dashed curve that approximates the time-dependent electronic energy gap. The expression used to calculate the approximate time-dependent electronic state separation can be found in the Supporting Information.

**Figure 3:**
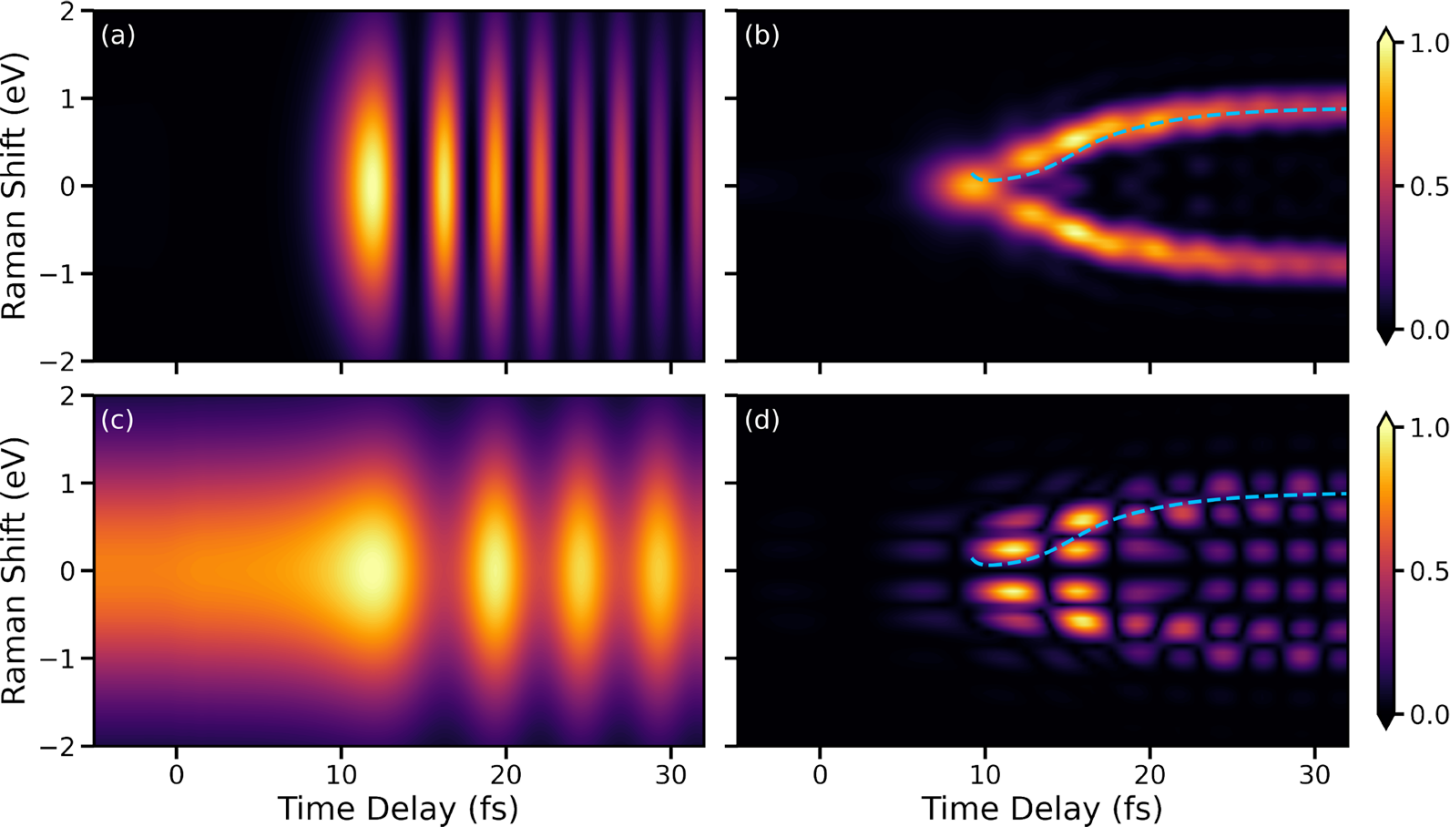
Comparison of homodyne-detected Raman spectra. (a) Electronic contribution to the Raman signal for a classical broadband pulse with a σ_f_ of 1.5 eV (σ_t_ = 0.44 fs). (b) Electronic contribution to the Raman signal constructed using entangled photons with a *T*_s_ of 10 fs and an ω_0_ – ω_i_ of 7.77 eV. The blue dotted curve represents an approximation for the time-dependent difference in energy between electronic states. (c) Raman spectrum for vibronic contributions created with a classical broadband pulse. (d) Processed Raman spectrum for vibrational and electronic contributions created using entangled photons. The entangled photon pairs are generated with a broadband SPDC pump with a pulse width (σ_f_) of 1.5 eV (σ_t_ = 0.44 fs).

The Raman spectra shown in panels a and b of Figure 3 include only the effect of the electronic coherences. However, to obtain a more realistic spectrum, the contributions from the vibrational coherences must also be taken into account (i.e., the diagonal elements of α̂). The full Raman signal with a classical broadband probe, including vibrational and electronic coherences, is shown in Figure 3c. The resulting signal presents a mixture of oscillatory features with a constant contribution. The constant component originates from the vibrational coherences that enter through ⟨α_00_(*t*)⟩ and ⟨α_11_(*t*)⟩. Similar to Figure 3a, the appearance of the CI can be resolved in the time domain but not in the Raman shift. For the Raman signal with entangled photons, the electronic contribution, similar to Figure 3b, is concealed by the strong vibrational contribution around 0 eV of the Raman shift. Therefore, the following equation will be used to process the Raman signal, as a new strategy to extract the desired features
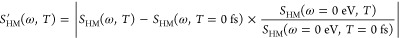
11The resulting signal obtained by using eq 11 is shown in Figure 3d. A strong peak appears around 10 fs and further shifts to higher Raman frequencies with an increase in the time delay. The signal follows the blue dashed curve, which represents the dynamic electronic energy gap. The oscillations of the signal along the blue dashed curve appear due to the entanglement between photons and originate from the two-photon wave function, Φ(ω′, ω_i_) (see eq 1). The two-photon wave function contains a sinc function that shows ripples along the frequency axis (ω′), which gives rise to the oscillations in the processed signal. The signal between ≈0.3 and −0.3 eV originates from the combination of the vibrational contribution and the sinc function in the two-photon wave function. Further analysis of the effect of the sinc function and the vibrational coherence to the processed signal can be found in the Supporting Information.

The presence of vibrational coherences in homodyne-detected spectra obscures the features of the electronic coherences, caused by the fact that the diagonal elements of α are larger in magnitude than the off-diagonal elements.^33,34^ The homodyne-detected signals rely solely on the photon number counts, and phase information imprinted in the emitted photons is lost. Such a phase, either stationary or dynamic, can be retrieved from interference with the local oscillator (LO) photons. This can be achieved by separating an extra path (the LO) from the s arm photons in the setup before the interaction with molecules, as depicted in Figure 1b. Interfering the LO photon with the emitted photons from molecules allows us to probe the phase change induced by the molecule–light interactions. This strategy may help to separate the time-evolving components from the static terms in the Raman signal arising from the phase difference between the LO and transmitted photons.

Figure 4 shows the heterodyne-detected Raman spectra obtained from eq 5, in which the electronic and vibrational components are involved. The phase difference between the signal and LO photons is set to ϕ = 0° for the simulations. Figure 4a shows the case for a classical broadband probe. Here, oscillations can be observed beginning at 10 fs, which are caused by the passage of the CI. However, the poor spectral resolution resulting from the broadband nature of the probe pulse conceals the time-evolving energy separation. In contrast, the entangled photon Raman signal, shown in Figure 4b, demonstrates a much better time–frequency resolution. The signal appears below 0.5 eV around 10 fs when α̃_01_(*t*) takes a nonvanishing value near the CI. For delays of >10 fs, the signal shifts toward higher Raman shifts, indicating an increasing level of electronic state splitting. Moreover, the peak envelope of the signal follows the time-evolving dynamics of electronic state splitting. The classical Raman signal for a narrowband probe is shown in Figure 4c. It represents a trade-off of time versus frequency resolution with respect to the time–bandwidth limit. Note that the heterodyne-detected spectra for classical pulses look similar to TRUECARS spectra,^34^ which can be used to probe electronic coherence near a CI. Compared to the presented heterodyne detection scheme that depends on a single photon to drive the Raman transitions, the TRUECARS method relies on two X-ray pulses that interact simultaneously with the molecule to drive stimulated Raman transitions between two electronic states.

**Figure 4:**
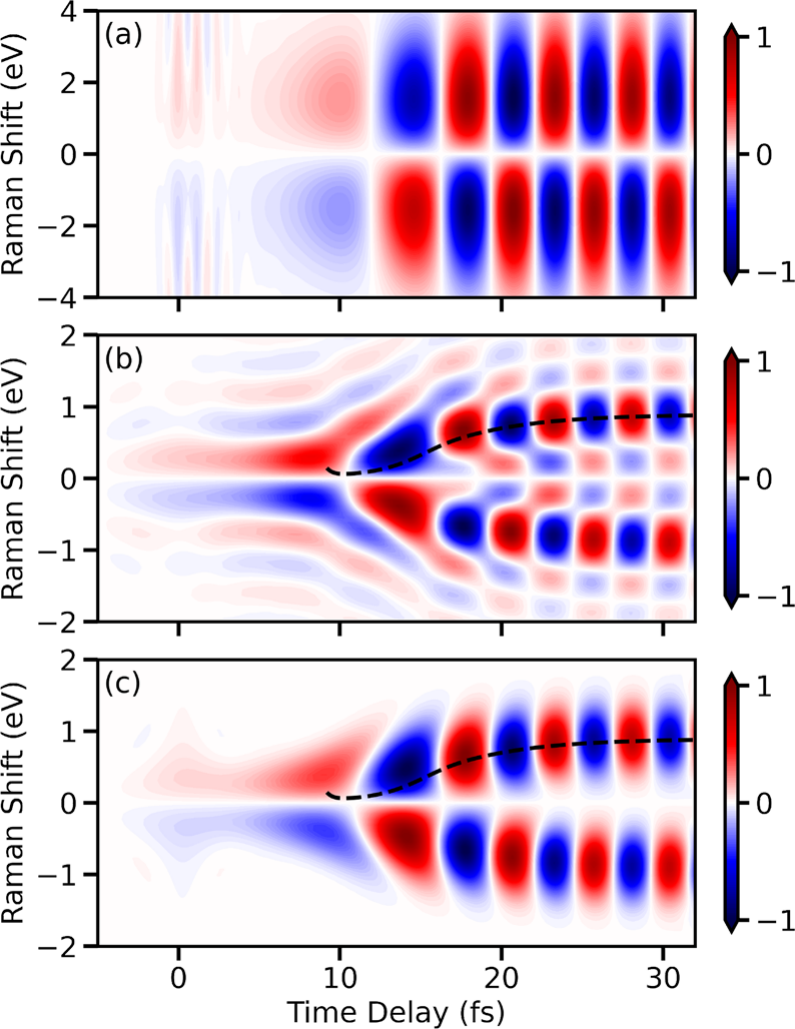
Comparsion of heterodyne-detected Raman signals. (a) Raman signal created using a classical broadband probe with a spectral width σ_f_ of 1.5 eV (σ_t_ = 0.44 fs). (b) Raman signal created using entangled photons with a *T*_s_ of 10 fs and an ω_0_ – ω_i_ of 7.77 eV. The entangled photons are generated using a SPDC pump with a spectral width σ_f_ of 1.5 eV (σ_t_ = 0.44 fs). The black dashed curve represents the time-dependent energy separation between the involved electronic states. (c) Raman signal generated by a classical narrow-band probe with a spectral width σ_f_ of 0.3 eV (σ_t_ = 2.19 fs).

In summary, our results demonstrate the capability of monitoring the ultrafast CI dynamics in molecules with a time–frequency resolution beyond that of classical light pulses. The heterodyne-detected Raman spectra produced by entangled photons yield a clearer signal of the electronic coherence created by the passage through the CI and the time-evolving energy gap can be read off directly from the Raman shift. The control over the phase enabled by the LO field allows visualization of the different components of the signal. In addition to the ϕ = 0° signal shown in Figure 4b where the electronic coherence component is clearly enhanced, the vibrational coherence component can be enhanced instead in the Raman signal when ϕ is set to 90°. Further details are provided in the Supporting Information.

We have presented a time-resolved Raman technique that makes explicit use of entangled photons and can directly map the time-evolving electronic energy gap in the vicinity of a CI. The technique is based, like previously proposed techniques,^20,40^ on the detection of electronic coherences, which are created when a molecule passes through a CI. Our simulations demonstrate that, in comparison with classical light, the entangled photons allow for a greater resolution in the time–frequency domain simultaneously. The use of entangled photons thus allows for lifting of the time–bandwidth restrictions imposed by classical light. The corresponding Raman spectra show a clear trace of the electronic energy gap in the time-resolved Raman shift. We have investigated both the homodyne-detected and heterodyne-detected signals. The homodyne-detected signal shows clear features of electronic and vibrational coherences. The heterodyne-detected signal, in contrast, allows for selective probing of the electronic or vibrational coherence features in the Raman signal. Here, the use of entangled photons yields an optimized spectral and temporal resolution while observing the electronic coherence generated near a CI, which cannot be attained using classical light.

The presented spectroscopic signal can be generalized to a broader class of coherent Raman spectroscopic signals. Some examples include entangled photon-stimulated Raman and multidimensional Raman processes. This would open a new frontier for studying the ultrafast processes in photochemistry, nanoplasmonics, and semiconducting heterostructures.

## Supporting Information Available

Details of photon entanglement, processed homodyne-detected Raman spectra, heterodyne-detected Raman spectra for ϕ = 90°, electronic state separation expression, and model system parameters with PES, couplings, and mixing angle figures (PDF)
