# Supplementary material for: Raman Spectroscopy of Conical Intersections Using Entangled Photons
Source: J Phys Chem Lett. 2024 Feb 13;15(7):2023–30. doi: 10.1021/acs.jpclett.3c02852 (PMC10895689; doi:10.1021/acs.jpclett.3c02852)
Supplement: Supplementary file 1 — jz3c02852_si_001.pdf [file jz3c02852_si_001.pdf]

Raman Spectroscopy of Conical Intersections using Entangled Photons

Deependra Jadoun and Markus Kowalewski*

Department of Physics, Stockholm University,

Albanova University Centre, SE-106 91 Stockholm, Sweden

Zhedong Zhang†

Department of Physics, City University of Hong Kong, Kowloon, Hong Kong SAR and

City University of Hong Kong, Shenzhen Research Institute, Shenzhen, Guangdong 518057, China

I. PHOTON ENTANGLEMENT

The entanglement between the photons generated using spontaneous parametric down-conversion (SPDC) is incorporated in the signal using the two-photon wave function given by,

$$\Phi(\omega_s,\omega_i) = E_0(\omega_s,\omega_i)\mathrm{sinc}\left[\left(\omega_s - \frac{\omega_0}{2}\right)\frac{T_s}{2} + \left(\omega_i - \frac{\omega_0}{2}\right)\frac{T_i}{2}\right] e^{i\left(\omega_s - \frac{\omega_0}{2}\right)\frac{T_s}{2} + i\left(\omega_i - \frac{\omega_0}{2}\right)\frac{T_i}{2}} \tag{S1}$$

where E_0 is the electric field for the SPDC pump with central frequency ω_0, and T_s (T_i) represents the delay between the SPDC pump and the photon in the s (i) arm of the setup. For a gaussian SPDC-pump, the two-photon wave function takes the following form [1],

$$\Phi(\omega_s,\omega_i) = e^{(\omega_s+\omega_i-\omega_0)^2/2\sigma_0^2} \times \mathrm{sinc}\left[\left(\omega_s - \frac{\omega_0}{2}\right)\frac{T_s}{2} + \left(\omega_i - \frac{\omega_0}{2}\right)\frac{T_i}{2}\right] \tag{S2}$$

where σ_0 is the pulse width of the SPDC-pump.

The entanglement between the photons is controlled by the delays T_s and T_i. Two-photon amplitudes calculated at two different delays using Eq. S2 are plotted in Fig. S1. For the delay T_s=0 fs, the two-photon wave function takes the shape of a gaussian function, whereas the increase in delay T_s gives a narrowband two-photon amplitude with ripples about the main peak at $\omega_s+\omega_i-\omega_0$=0 eV.

* Correspondence email address: markus.kowalewski@fysik.su.se

† Correspondence email address: zzhan26@cityu.edu.hk

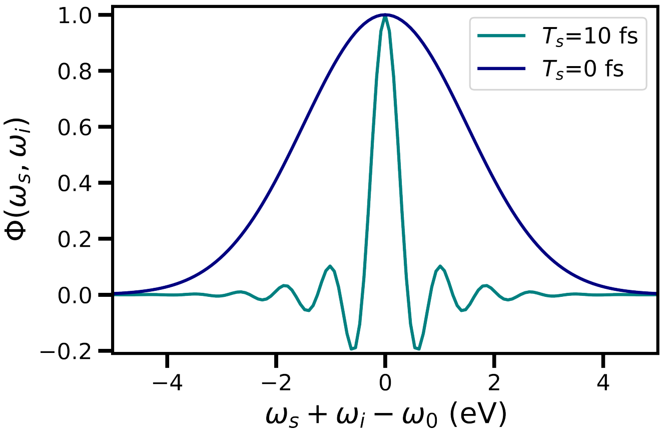

Figure S1. Two-photon amplitudes for a gaussian SPDC-pump with the pulse width of σ_0=1.5 eV are shown.

The degree of entanglement can be studied using the entanglement entropy of the photon pairs using the following expression,

$$E(\Phi) = -\sum \lambda_n^2 ln(\lambda_n^2) \tag{S3}$$

where λ_n represents the normalized set of eigenvalues of the two-photon states following the singular value decomposition with $\sum \lambda_n^2 = 1$. The two-photon wave function following the singular value decomposition can be written as [2],

$$\Phi(\omega_s, \omega_i) = \sqrt{A} \sum \lambda_n \psi_n^*(\omega_s) \phi_n^*(\omega_i) \tag{S4}$$

where $\{\phi_n\}$ and $\{\psi_n\}$ represent the Schmidt modes and form orthonormal bases. The entanglement entropy for σ_0=1.5 eV is shown in Fig. S2. As can be seen in Fig. S2, the entanglement entropy increases with increasing delays of the entangled photons (T_s and T_i). The entanglement entropy falls when the entanglement time, $T_e = T_s - T_i$, increases, and the maximum entanglement is achieved at $T_s = T_i$.

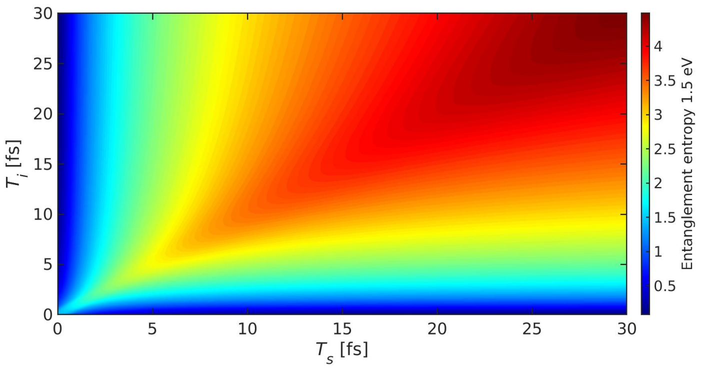

Figure S2. The entanglement entropy for σ_0=1.5 eV.

II. ADDITIONAL SPECTRA

A. Processed Homodyne Raman Spectra

The homodyne Raman signal for vibronic contributions constructed using entangled photons is dominated by vibrational contributions. The signal is processed using Eq. 15 in the main text, which leads to the signal shown in Fig. 3(d). The effect of vibrational contributions and the ripples around the main peak in the *sinc* function in $\Phi(\omega', \omega_i)$ (see Fig. S1) is analyzed here. The effect of the ripples around the main peak in the *sinc* function can be filtered by flattening all the side peaks in the *sinc* function. Figure S3 shows the spectra where the flattened *sinc* is used in $\Phi(\omega', \omega_i)$. The signal for a flattened *sinc* function corresponding to the signal in Fig. 3(d) is shown in Fig. S3(a). As can be seen in the signal that follows the blue dashed curve, there are no oscillations. When the strength of the vibrational elements $(\langle\alpha_{00}(t)\rangle + \langle\alpha_{11}(t)\rangle)$ is reduced by a factor of 10, the signal at the Raman shift of 0 eV starts to become weak Fig. S3(b). A similar trend can be seen in Fig. S3(c) where the vibrational elements is reduced by the factor of 100, and only the signal for the electronic Raman transitions is visible.

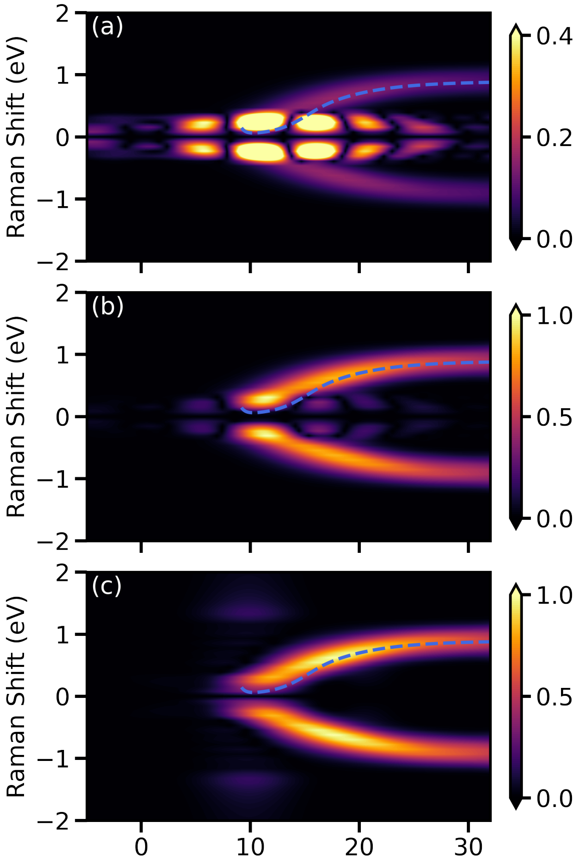

Figure S3. Processed homodyne detected spectra for flattened *sinc* function. (a) Signal similar to Fig. 3(d). (b) The signal for a downscaled vibration contribution, $(\langle\alpha_{11}\rangle + \langle\alpha_{00}\rangle)/10$. (c) The signal for a downscaled vibrational contribution, $(\langle\alpha_{11}\rangle + \langle\alpha_{00}\rangle)/100$.

B. Heterodyne-Detected Raman Spectra

As stated in the main text, the change in the phase of the local oscillator (LO) and signal photons helps study different features in the Raman signals. Figure 4 in the main text shows the Raman spectra with the zero phase difference between the LO and signal photons, and only features corresponding to the electronic contribution are visible. The Raman spectra for a phase difference of 90° between the LO and signal photons are shown in Fig. S4. As can be seen in the spectra, the vibrational contributions to the Raman signal dominate over the electronic transition features.

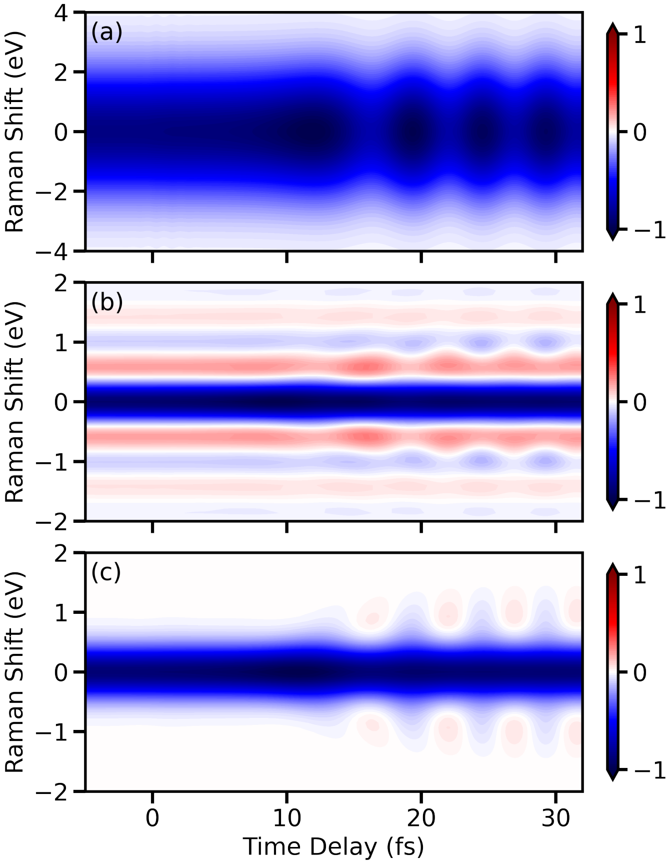

Figure S4. Heterodyne-detected Raman signals for vibronic contributions with 90° phase difference between the local oscillator (LO) and signal photons. (a) Raman signal generated by a classical broadband probe with a spectral width of σ_f=1.5 eV. (b) Raman signal produced by entangled photons with T_s=10 fs generated using a SPDC-pump with a spectral width of σ_f=1.5 eV. The black-dashed curve represents the time-dependent energy separation. (c) Raman signal generated by a classical narrowband probe with a spectral width of σ_f=0.3 eV.

III. TIME-DEPENDENT ELECTRONIC STATE SEPARATION

The time-dependent energy difference between two electronic states can be approximated using the electronic coherence generated via the CI. The following expression is used to calculate the

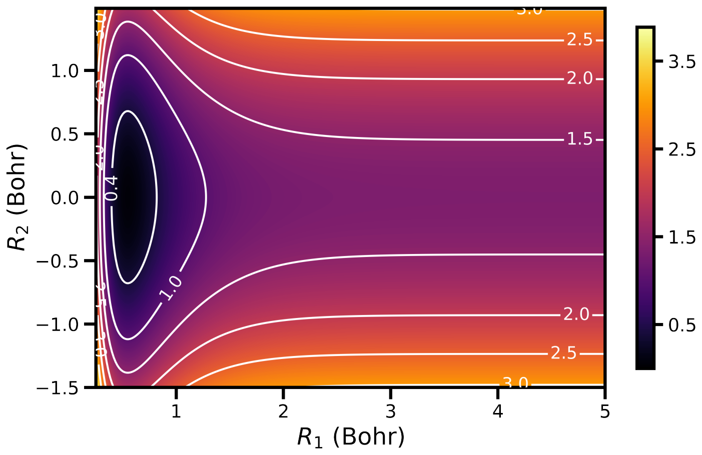

Figure S5. The potential energy surface (PES) for the valence state V_0 is shown here. The energy (in eV) is represented on the color bar.

time-dependent electronic state separation,

$$\Delta V(t) = \frac{\int \mathrm{d}R_1 \mathrm{d}R_2 \Big| V_1 - V_0 \Big| \Big| \psi_0(t)\psi_1(t) \Big|}{\int \mathrm{d}R_1 \mathrm{d}R_2 \Big| \psi_0^*(t)\psi_1(t) \Big|} \tag{S5}$$

IV. MODEL SYSTEM

Atomic units ($\hbar = e = m_e = 4\pi\epsilon_0 = 1$) are used hereafter until stated otherwise. The following expressions were used to construct the diabatic potential energy surfaces and the diabatic couplings as a function of reaction coordinates R_1 and R_2,

$$\begin{aligned} V_0 &= \frac{0.2 + 2e^{-6(R_1-0.1)} - e^{-2.5(R_1-0.1)}}{4} + \frac{R_2^2}{36} - \frac{0.0147}{27.211} \\ V_1 &= \frac{0.05 + e^{-2R_1}}{3} + \frac{R_2^2}{36} \\ C_g &= 0.001e^{-2(R_1-1.3545)^2}(1 - e^{-5|R_2|})\left(1 - \left|\frac{R_2 - |R_2|}{|R_2|}\right|\right) \\ C_{01} &= \frac{C_g}{80 \times \max(C_g)} \end{aligned} \tag{S6}$$

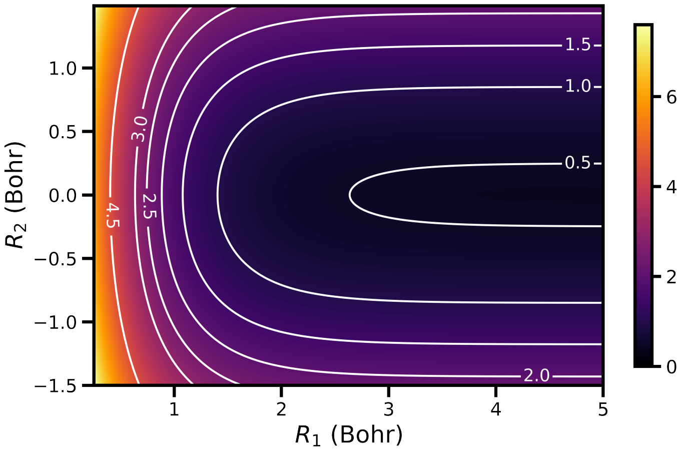

Figure S6. The potential energy surface (PES) for the valence state V_1 is shown here. The energy (in eV) is represented on the color bar.

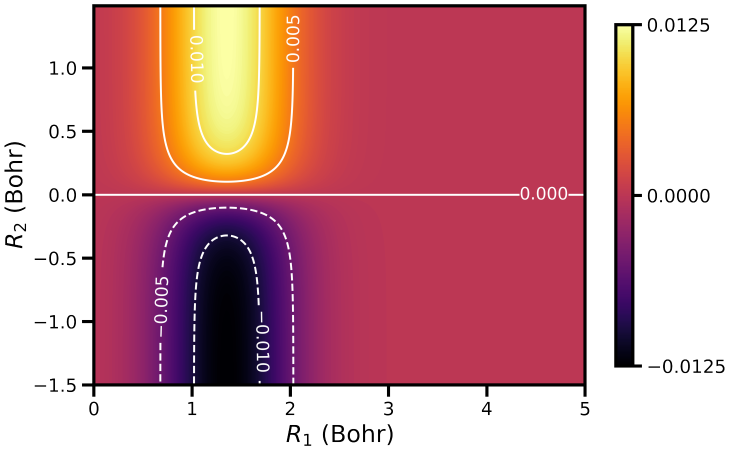

Figure S7. The diabatic coupling terms between the valence states V_0 and V_1 are shown here. The dashed contours represent negative values, and the coupling strength is represented in atomic units on the color bar.

where V_0 and V_1 represent diabatic electronic states, and C_{01} represents diabatic couplings between the electronic states. The potential energy surface (PES) of the V_0 state is shown in Fig. S5, the PES of the V_1 state is shown in Fig. S6, and the diabatic couplings are shown in Fig. S7. For the diabatic to adiabatic transformation to visualize the conical intersection (CI), the mixing angle

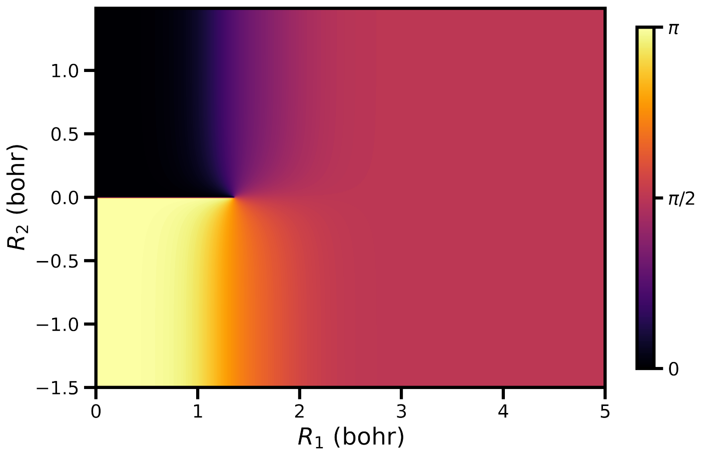

Figure S8. The mixing angle that can be used for diabatic to adiabatic transformation is shown here. The angle is represented on the color bar. The emergence of the Berry phase can be seen.

should be calculated using the following expression,

$$\theta(R_1, R_2) = \frac{1}{2}\tan^{-1}\left(\frac{2C_{01}(R_1, R_2)}{V_1(R_1, R_2) - V_0(R_1, R_2)}\right) \tag{S7}$$

The mixing angles calculated using the above expression are shown in Fig. S8.

Quantum dynamics in the model system is initiated using a pump-pulse, which excites the nuclear wave packet from the V_0 state minimum to the Franck-Condon point on the V_1 state. Snapshots of the moduli of the nuclear wave functions depending on the reaction coordinates R_1 and R_2 are shown in Fig. S9 for different pump-probe delays. Figures S9(a) and (b) show the moduli of the nuclear wave functions at the pump-probe delay of -2.5 fs for the V_0 and V_1 states, respectively. The negative pump-probe delay indicates that the pump-pulse is yet to interact, and hence the nuclear wave packets are present only in the V_0 state, as shown in Fig. S9(a). Following the pump-pulse interaction, both the valence states are populated, as shown in Figs. S9(c) and (d), which show the moduli of the nuclear wave functions at the delay of 6 fs for the V_0 and V_1 state, respectively. The passage through the CI gives rise to the population exchange between the two electronic states, as shown in Figs. S9(e) and (f) for the pump-probe delay of 20 fs. The nuclear wave packet in the V_1 state (Fig. S9(f)) goes through the CI, and transfers the population to the V_0 state, as shown in Fig.

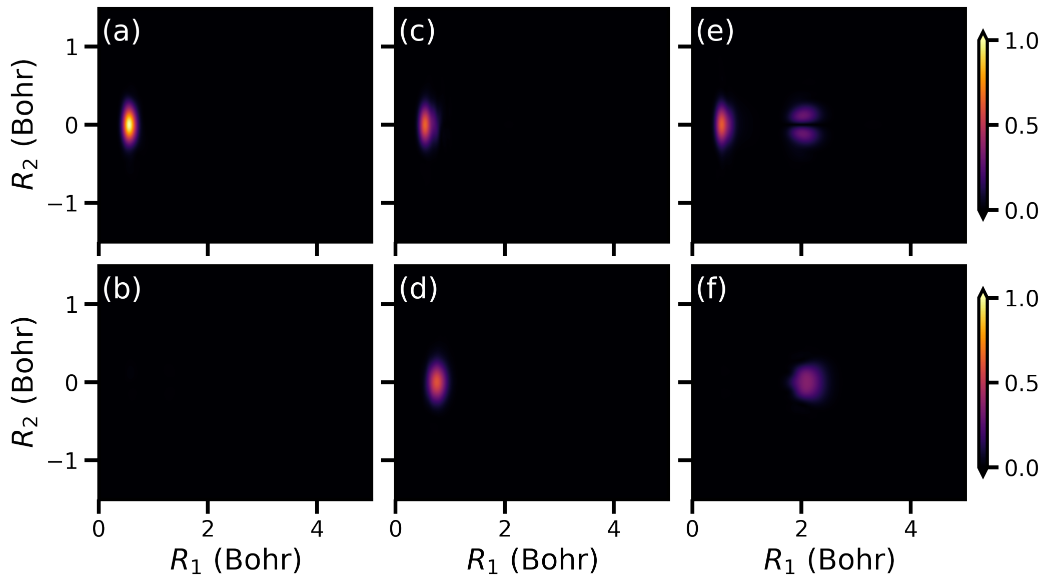

Figure S9. Nuclear coordinate dependence of the modulus of nuclear wave functions for the valence states are shown at different pump-probe delays (T). (a),(c),(e) Nuclear wave packets ($|\psi_0|$) for the V_0 state at T=-2.5 fs, T=6 fs, and T=20 fs, respectively. (b),(d),(e) Nuclear wave packets ($|\psi_1|$) for the V_1 state are shown for the delays T=-2.5 fs, T=6 fs, and T=20 fs, respectively. The strength of the wave packets is normalized with respect to the peak strength in (a) for the sake of comparison.

S9(e). The node along the R_1 coordinate in Fig. S9(e) indicates the node in the diabatic couplings at the CI. The strengths of the absolute nuclear wave functions are normalized with respect to the wave packet in Fig. S9(a).

V. COMPUTATIONAL DETAILS

A grid with 300 $\times$ 300 points is used for the construction of the potential energy surfaces of the model system. Our in-house code QDng is used for the wave packet propagation on the potential energy surfaces, and the Arnoldi method is used to solve the time-dependent Schrödinger equation[3]. The Fourier transform method is used to calculate the second-order derivative for the calculation of the kinetic energy of wave functions. The step-size used in the quantum dynamics of the nuclear wave packets in electronic states is $\approx$48 as. A pump-pulse with the field strength of A_P=10.28 GV/m,

frequency of ω_P=3.6 eV, and pulse width σ_P=1 fs is used for the excitation of the molecular model system. A constant transition dipole moment (μ_P) with the value of 1 a.u. is used. The time-dependent expectation value of the polarizability operator ($\langle\alpha(t)\rangle$) in Eq. 3 and Eq. 5 in the main text is calculated using time-dependent wave functions obtained from the quantum dynamics simulations.

[1] Asban, S.; Mukamel, S. Distinguishability and "which pathway" information in multidimensional interferometric spectroscopy with a single entangled photon-pair. *Sci. Adv.* **2021**, *7*, eabj4566.

[2] Dorfman, K. E.; Schlawin, F.; Mukamel, S. Nonlinear optical signals and spectroscopy with quantum light. *Rev. Mod. Phys.* **2016**, *88*, 045008.

[3] Arnoldi, W. E. The principle of minimized iterations in the solution of the matrix eigenvalue problem. *Q. Appl. Math.* **1951**, *9*, 17–29.
